# Myocardial Function Maturation in Very-Low-Birth-Weight Infants and Development of Bronchopulmonary Dysplasia

**DOI:** 10.3389/fped.2019.00556

**Published:** 2020-01-17

**Authors:** Paula Méndez-Abad, Pamela Zafra-Rodríguez, Simón Lubián-López, Isabel Benavente-Fernández

**Affiliations:** ^1^Department of Neonatology, Puerta del Mar University Hospital, Cádiz, Spain; ^2^Department of Pediatrics, Institute for Research and Innovation in Biomedical Sciences (INiBICA), Cádiz, Spain

**Keywords:** bronchopulmonary dysplasia, preterm infants, myocardial function, N-terminal pro B type natriuretic peptide, tissue doppler imaging, targeted neonatal echocardiography, biomarkers, tricuspid annular plane systolic excursion

## Abstract

**Background:** Myocardial function in very-low-birth-weight infants (VLBWIs) develops during early postnatal life, but different patterns of temporal evolution that might be related to the development of bronchopulmonary dysplasia (BPD) are not completely understood.

**Methods:** A prospective cohort study including VLBWIs admitted to our NICU from January 2015 to 2017 was conducted. Plasma N-terminal pro B type natriuretic peptide (NTproBNP) levels were measured, and echocardiograms were performed at 24 and 72 h of life and weekly thereafter until 36 weeks of postmenstrual age (PMA). We measured the tricuspid annular plane systolic excursion (TAPSE) by M-mode; the lateral tricuspid E', A', and S' waves; and the myocardial performance index (MPI) by tissue doppler imaging (TDI). The subjects were divided into non-BPD and BPD groups.

**Results:** We included 101 VLBWIs. The TAPSE and E', A', and S' waves increased while MPI-TDI decreased over time. Birth gestational age (GA) and postnatal PMA impacted these parameters, which evolved differently in those who developed BPD compared to those in the non-BPD group. The NTproBNP levels at 14 days of life and different echocardiographic parameters were associated with the development of BPD in different multivariate models.

**Conclusion:** TAPSE and TDI values depend on GA and PMA and follow a different temporal evolution that is related to the later development of BPD. Combined biochemical and echocardiographic biomarkers can help identify which VLBWIs are at higher risk of developing BDP.

## Introduction

Bronchopulmonary dysplasia (BPD) is a severe complication of prematurity that impacts the survival and development of very-low-birth-weight infants (VLBWIs) (1). The incidence of BPD in VLBWIs remains stable at ~25% despite improvements in neonatal care and the survival rate of this population (1). VLBWIs with BPD have increased lengths of hospital stay and greater morbidity and mortality (2). There is an increasing body of research aiming to identify early markers associated with BPD development in VLBWIs that could help identify high-risk infants who would benefit from potential preventive strategies (3, 4).

Targeted neonatal echocardiography (TnEcho) has expanded in neonatal intensive care units as an additional tool in the hemodynamic assessment of newborns (5). Recently, there has been growing research interest in the potential role of conventional and emerging echocardiographic modalities, such as tissue Doppler imaging (TDI), in the assessment of newborn and VLBWIs cardiopulmonary morbidities including patent ductus arteriosus (PDA) and BPD (6). Normative values of TnEcho parameters have been described for children (7) and healthy newborns (8). Nevertheless, myocardial function in the VLBWIs is known to be different from that of the term newborn (9), but the influence of postnatal life on VLBWIs myocardial maturation and how it differs in those who later develop BPD are not completely understood (10).

However, biochemical biomarkers, such as N-terminal pro B type natriuretic peptide (NTproBNP), have been associated with neonatal morbidities, such as persistent pulmonary hypertension (11) and PDA (12). In infants with BPD, NTproBNP is a suggested marker of pulmonary hypertension (3, 13). NTproBNP levels in the first month of life increase in VLBWIs who develop BPD (12, 14), as we have previously shown (15), and are related to the degree of BPD (16, 17).

This study aims to evaluate the temporal evolution of VLBWI myocardial function related to gestational age (GA) and postmenstrual age (PMA) and to explore the potential role of right ventricle (RV) TDI-derived measurements, tricuspid annular plane systolic excursion (TAPSE) and NTproBNP together to detect early signs of myocardial dysmaturation in infants who develop BPD.

## Materials and Methods

### Subjects

This was a prospective observational study that included VLBWIs admitted to the Neonatal Intensive Care Unit of the University Hospital Puerta del Mar (Cadiz, Spain) from January 2015 to 2017. The institutional research ethics committee approved the study, and written informed consent was obtained from the parents in accordance with the Declaration of Helsinki. Patients with birth weights ≤1,500 g and/or a GA of ≤32 weeks were included. Exclusion criteria were congenital heart disease (except patent foramen ovale or atrial septal defect, ventricular septal defect < 2 mm, or PDA); genetic syndrome or major congenital malformations; death in the first week of life; and a lack of informed consent from the parents.

We defined a subgroup of VLBWIs that we considered “healthy VLBWIs” to define the normal values of the different echocardiographic parameters. We considered “healthy VLBWIs” those with an adequate weight for GA (18–20), with no hemodynamically significant PDA (21, 22) (defined as PDA <1.5 mm in any TnEcho or < 1 mm after 32 weeks PMA) and no BPD (23). The myocardial maturation of preterm infants who are born small for gestational age has been suggested to be affected from intrauterine life and this could be reflected in a worse cardiac function detected from birth to childhood (24). Preterm infants who develop BPD may have an increased RV afterload secondary to an incipient vascular lung disease, on the other hand, a PDA would increase the left ventricle preload, and could also worsen myocardial function (18).

### Measurements

The following variables were collected: GA, birth weight, sex, small for gestational age (SGA), premature rupture of membranes, chorioamnionitis, 5 min Apgar score, clinical risk index for babies (CRIB), prenatal steroid use, hyaline membrane disease, sepsis, postnatal steroids, need of inhaled nitric oxide (iNO), severe intraventricular hemorrhage with or without parenchymal infarction, white matter injury, PDA, retinopathy of prematurity, and necrotizing enterocolitis. The need for and duration of mechanical ventilation were also recorded. The included VLBWIs were classified into two groups according to the presence or absence of BPD defined as the need for oxygen or respiratory support at 36 weeks of PMA (25). BPD was further classified in moderate or severe BPD: moderate BPD was considered if <30% oxygen was needed and severe BPD if more than 30% oxygen was needed or the infants was on respiratory support (25).

**Figure 1:**
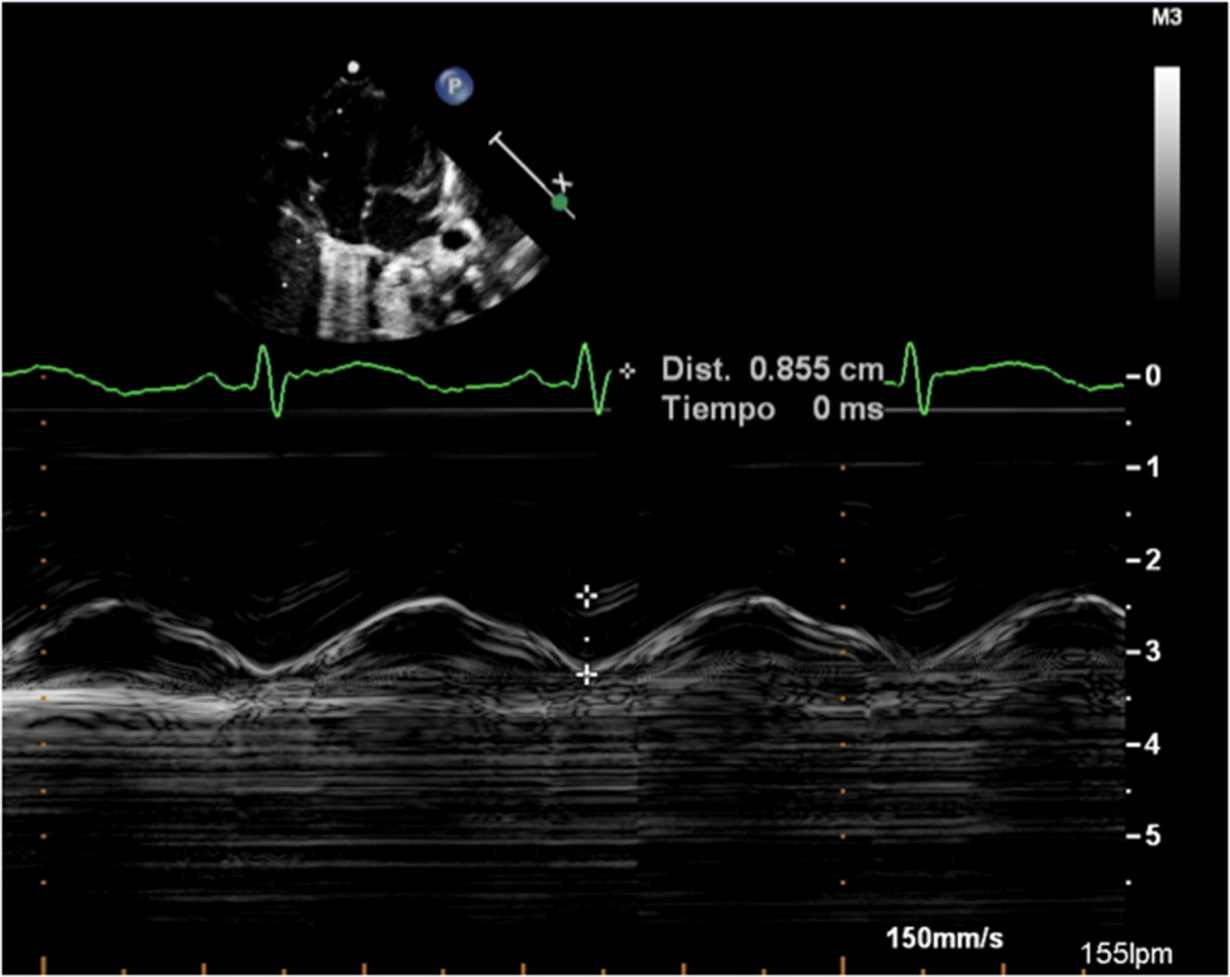
Measurement of the tricuspid annular plane systolic excursion (TAPSE).

TDI was performed at the lateral ring of the tricuspid valves from an apical four chamber view using a sample volume size of 1.5 mm and maintaining an angle of insonation of <20° with respect to the orientation of the ventricular wall. No angle correction was made, and gains were minimized to allow a clear signal from the tissue (26). Early diastolic (E′), late diastolic (A′), and systolic (S′) velocities were measured. The myocardial performance index measured by TDI (MPI-TDI) was calculated as (a–b)/b, where “a” is the time interval from the end of wave A′ to the beginning of wave E′ and “b” is the interval between the beginning and end of the S′ wave. TAPSE was measured by M-mode echocardiography with the scan line passing through the lateral face of the tricuspid ring, maintaining vertical alignment with the apex (5) (Figure 1).

To measure NTproBNP plasma levels, 0.5 ml of blood sample was collected in ethylenediaminetetraacetic acid (EDTA), transported at room temperature and processed immediately for analysis. NTproBNP levels were calculated by means of the electroluminescence immunoassay kit (ECLIA) with the Elecsys proBNP II test (Roche Diagnostics).

### Protocol

Echocardiograms were performed in the first 24 and 72 h of life and then weekly until 36 weeks of PMA. The exam was performed with extreme care and minimization of the study time. The echocardiographic evaluation was not performed if the VLBWI was unstable, and the procedure was stopped if the patient's condition deteriorated. Two neonatologists with specific training in neonatal echocardiography performed the studies using a Philips iE33 with an 8–3 Hz transducer according to the recommendations of the American Society of Echocardiography (5). The interobserver and intraobserver reproducibility was previously analyzed and reported by our group (27). Electrocardiographic records were taken simultaneously. The studies were digitally stored and analyzed offline. At least three consecutive beats were recorded in sinus rhythm, and the mean values were recorded.

Following the same schedule as the echocardiographic exam, but only if a blood test was clinically indicated, NTproBNP levels were measured.

### Data Analysis

The quantitative variables are described as the mean and standard deviation (SD) or median and range according to their distributions. Qualitative variables are expressed as the frequency and percentage. The bivariate analysis was performed with parametric or non-parametric techniques after testing for normality.

As a first approach, we studied the temporal evolution of the TAPSE, E', A, and S' velocities of the RV in the “healthy VLBWI” subgroup, related to GA and PMA. GA was divided into three groups: 25–27^+6^, 28–30^+6^, and 31–32^+6^ weeks. The measurements of the first day of life (DOL) were considered those related to GA and were compared to those at a similar PMA to study the influence of extrauterine life on the evolution of these velocities. Considering the first DOL as the transversal study for each week of gestation, the longitudinal study was performed excluding this first DOL TnEchos and related to the corrected age (PMA). We performed linear regression to determine the associations of all the studied parameters with GA and PMA.

Returning to the whole study sample, we then performed mixed-effects logistic regression analysis to study the relationship of the mentioned velocities longitudinally over time with the later BPD development, adjusting for repeated TnEchos in each patient. To test which of the waves should be included in the multivariate model, we first included all of the measured parameters, NTproBNP, GA and PMA, and computed all possible combinations of the independent variables, allowing nonhierarchical models. Mallow's Cp, Akaike Information Criteria (AIC), Schwarz Bayesian Criterion (BIC), R-squared (R^2^) and adjusted R^2^ (R^2^Adj) were computed for each subset. Based on these goodness-of-fit parameters, the theoretical background and parsimony, we selected two models for further post-estimation analysis and validated them internally (cross-validation) and externally (shrinkage).

## Results

### Study Population

During the recruitment period, 120 VLBWIs were admitted to the NICU. We excluded 9 (7.5%) that died in the first week of life, 2 (1.6%) were excluded because they had chromosomal diseases, and one (0.86%) was excluded due to a congenital heart defect. A total of 101 subjects with a median birth weight of 1,115 g (580–1,680 g) and a median GA of 29 weeks (25–32 weeks) were included as the final study sample. Fifteen patients met the diagnostic criteria for BPD, with five cases of moderate BPD and 10 cases of severe BPD. Four participants died during admission, one of them with severe BPD. The patients who developed BPD had lower GAs and birth weights and had severe retinopathy of prematurity, hyaline membrane disease and PDA in greater proportion than the non-BPD patients. They also had a higher CRIB score, need of postnatal steroids and were ventilated for a longer period (Table 1). A total of 483 echocardiograms were performed. Missing values ranged from 2.7% (TAPSE and E' wave) to 6.42% (S' wave). A total of 223 blood samples were analyzed.

**Table 1:** Perinatal variables in no-BPD and BPD groups.

	**Non-BPD** **(*n* = 86)**	**BPD** **(*n* = 15)**	**Total** **(*n* = 101)**	***p***
GA	29.13 ± 1.79	27.27 ± 1.3	29 (25–32)	0.0002*
Weight (g)	1,200 (600–1,680)	850 (580–1,400)	1,150 (580–1,680)	0.0001*
Sex, male	45 (52.33%)	11 (72.3%)	56 (55.45 %)	0.13
SGA	9 (10.47%)	4 (26.67%)	13 (12.87%)	0.084
Apgar 5	8 (4–10)	7 (4–8)	7 (4–10)	0.002*
CRIB	1 (0–7)	5 (1–12)	1 (0–12)	0.0001*
Chorioamnionitis	12 (13.95%)	4 (26.67%)	16 (15.84%)	0.2
Prenatal steroids (≥1 dose)	72 (83.72%)	14(93.33%)	86 (85.15%)	0.33
Cesarean section	72 (83.72%)	12 (80%)	84 (83.17%)	0.72
Early onset sepsis	1 (1.16%)	0	1 (0.99%)	0.67
Late onset sepsis	12 (13.95%)	5 (33.33%)	17 (16.83%)	0.064
Hyaline membrane disease	51 (59.3%)	13 (86.67%)	64 (63.37%)	0.04
Mechanical ventilation	53 (61.63%)	14 (93.33%)	67 (67.6%)	0.016*
Days of mechanical ventilation	3 (0–39)	40 (0–173)	14.16 ± 29.87	0.0001*
Postnatal steroids	1 (1.16%)	3 (20%)	4 (3.96%)	0.001
iNO	4 (4.65%)	2 (13.33%)	6 (5.94)	0.189
PDA	37 (43.02%)	12 (80%)	49 (48.5%)	0.011*
Severe IVH	2 (2.33%)	1 (6.67%)	3 (2.97%)	0.39
White matter injury	2 (2.33%)	1 (6.67%)	3 (2.97%)	0.39
Severe ROP	7(8.14)	5 (33.33)	12 (%)	0.016*
NEC	3 (3.49%)	0	3 (3.49%)	1.00

GA, Gestational age (weeks); BPD, bronchopulmonary dysplasia; SGA, small for gestational age; iNO, inhaled nitric oxide; PDA, patent ductus arteriosus; Severe IVH, Grade III intraventricular hemorrhage or parenchymal hemorrhagic infarction; Severe ROP, retinopathy of prematurity ≥ grade 2; NEC, Necrotizing enterocolitis; p, Statistical significance (<0.05) between both groups. *p < 0.05.

### Evolution of TAPSE and TDI in the “Healthy VLBWI” Subgroup

In the “healthy VLBWI” subgroup, we included 63 patients: 9 (14.3%) in the 25–27^+6^ week group, 39 (61.9%) in the 28–30^+6^ week group and 15 (23.8%) in the 31–32^+6^ week group. The median birth weight was 1,220 g (600–1,680). Fifteen patients needed oxygen at 28 DOL but were given room air at 36 PMA. The perinatal characteristics of this subgroup are summarized in Table 2.

**Table 2:** Perinatal variables in the “healthy VLBWI” group.

	**25–27 + 6 GA** **(*n* = 9)**	**28–30 + 6 GA (*n* = 39)**	**31–32 GA (*n* = 15)**	**Total** **(*n* = 63)**
GA	25.78 ± 0.83	29.03 ± 0.74	31.27 ± 0.59	29.10 ± 1.80
Weight (g)	900 (600–1,300)	1,200 (950–1,570)	1,375 (1,215–1,680)	1,220 (600–1,680)
Sex, male	4 (44.4%)	22 (56.41%)	10 (66.67%)	36 (57.14%)
Apgar 5	7 (4–8)	8 (4–10)	8 (6–10)	8 (4–10)
CRIB	2 (1–7)	1 (0–6)	0 (0–1)	1 (0–7)
Chorioamnionitis	1 (11%)	6 (15%)	3 (20%)	10 (16%)
Prenatal steroids (≥1 dose)	8 (89%)	31 (80%)	14 (93%)	52
Cesarean section	7 (77%)	30 (76.9%)	12 (80%)	49 (78%)
Early onset sepsis	0	0	0	0
Late onset sepsis	3 (33%)	4 (10%)	1 (6.6%)	8 (12.4%)
Hyaline membrane disease	6 (66.67%)	27 (69.23%)	3 (20%)	36 (57.14%)
Mechanical ventilation	8 (88.89%)	26 (66.67%)	3 (20%)	37 (58.73%)
Days of mechanical ventilation	14.33 ± 13.14	5.79 ± 7.87	0.87 ± 1.81	5.84 ± 8.80
Postnatal steroids	0	1 (2.56)	0	1 (1.59%)
iNO	1 (11.11%)	2 (5.13%)	0	3 (4.76%)
Mild BPD	5 (55.5%)	10 (25.6%)	0	15 (23.8%)
Severe IVH	1 (11%)	1 (2.5%)	0	2 (3.1%)
White matter injury	1 (11%)	1 (2.5%)	0	2 (3.1%)
Severe ROP	1 (11%)	0	0	1 (6.2%)
NEC	1 (11%)	0	0	1 (1.5%)

*GA, Gestational age (weeks); iNO, inhaled nitric oxide; BPD, bronchopulmonary dysplasia; Severe IVH, Grade III intraventricular hemorrhage or parenchymal hemorrhagic infarction; Severe ROP, retinopathy of prematurity ≥ grade 2; NEC, Necrotizing enterocolitis*.

**Figure 2:**
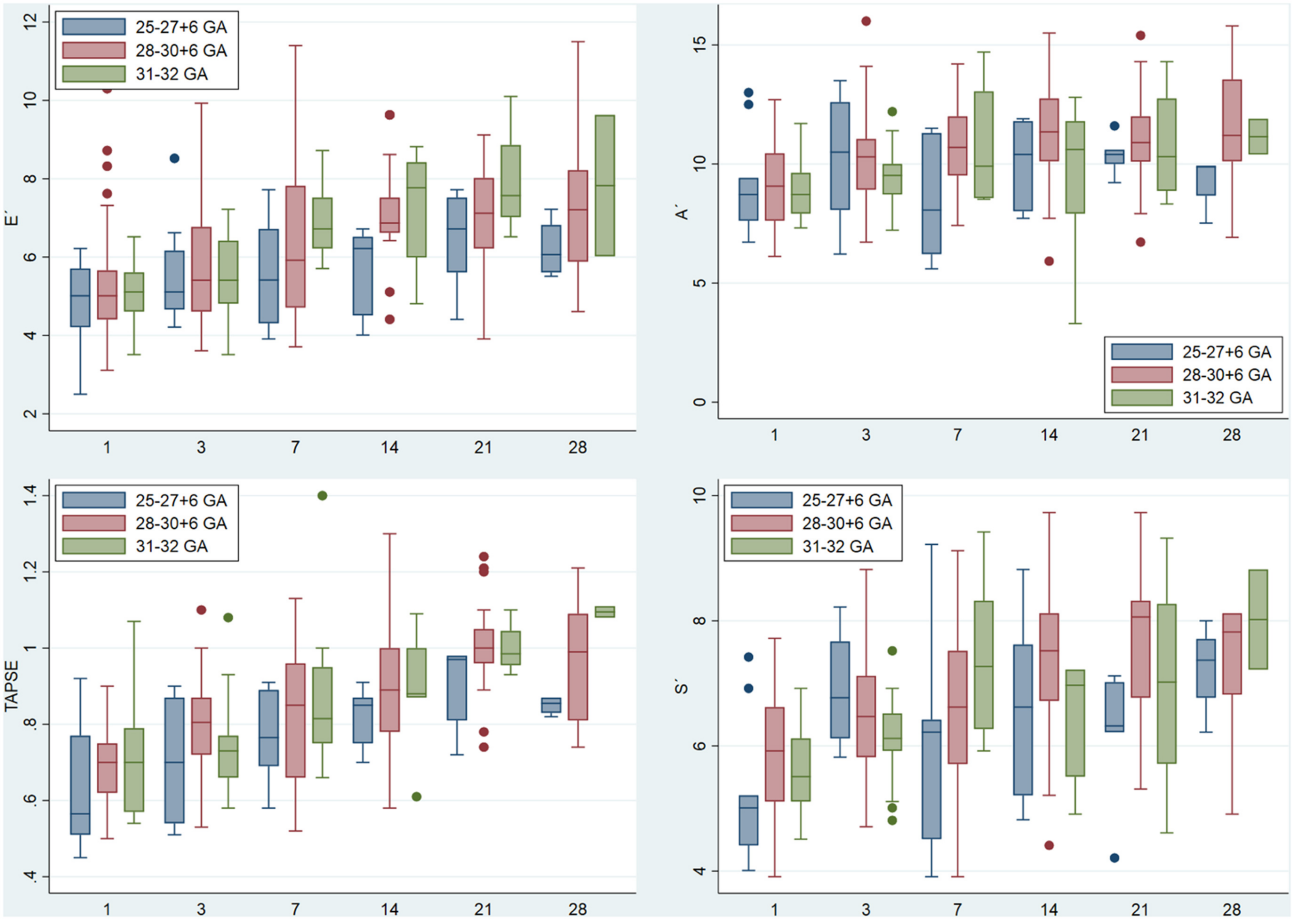
Right ventricle echocardiographic parameters in the first month of life by gestational age. y-axis: E', Lateral tricuspid early diastolic velocity by tissue doppler (cm/s); A', Lateral tricuspid late diastolic velocity by tissue doppler (cm/s); TAPSE, Tricuspid annular plane systolic excursion (cm). S', Lateral tricuspid systolic velocity by tissue doppler (cm/s). GA, Gestational age (weeks); x-axis, days of life.

**Figure 3:**
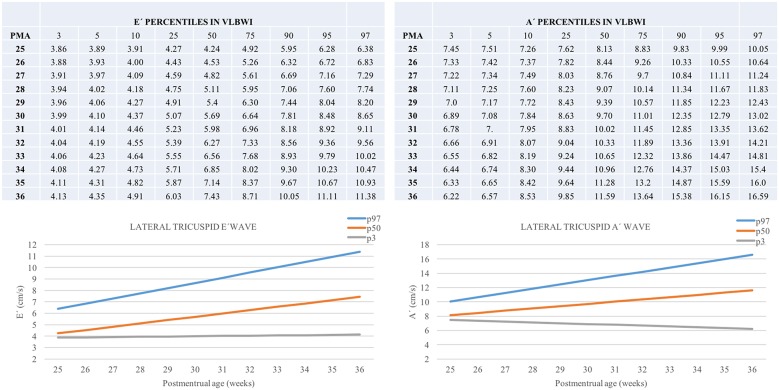
Percentiles of lateral tricuspid E' and A' in VLBWI by PMA. E', Lateral tricuspid early diastolic velocity by tissue doppler (E'); A', Lateral tricuspid late diastolic velocity by tissue doppler; PMA, postmenstrual age (weeks). The longitudinal study was performed excluding this first day of life TnEchos and related to the corrected age.

**Figure 4:**
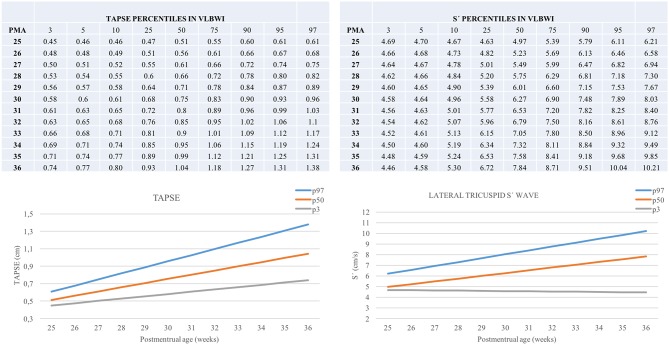
Percentiles of TAPSE and lateral tricuspid S' in VLBWI by PMA. TAPSE, Tricuspid annular plane systolic excursion; (S'), Lateral tricuspid systolic velocity by tissue doppler; PMA, postmenstrual age (weeks). The longitudinal study was performed excluding this first day of life TnEchos and related to the corrected age.

TAPSE, E', A', and S' increased, and MPI-TDI decreased with chronological age. All parameters were lower in the group with the lowest GA (Figure 2, Table S1). We described the predicted percentiles according to PMA (Figures 3, 4).

As TAPSE differed according to GA compared to PMA [0.83 (95% CI 0.78–0.88) vs. 0.91 (95% CI 0.88–0.94); *p* = 0.002], we predicted TAPSE with both GA and PMA, obtaining the equation TAPSE (cm/s) = −0.36–0.03xGA + 0.06xPMA (R^2^ Adj = 0.54, *p* = 0.0001). Similarly, an estimation of the RV velocities and MPI measured by TDI is described in Table 3. The observed and predicted values of these are presented in the Table S2.

**Table 3:** Linear regression equations of the right ventricle TnEcho parameters and TAPSE by gestational age and postmenstrual age.

Predicted TAPSE = −0.359 −0.030 GA + 0.065 PMA
Predicted E' = −1.055 −0.158 GA + 0.375 PMA
Predicted A' = 3.110 −0.228 GA + 0.434 PMA
Predicted S' = 2.446 −0.1921 GA + 0.307 PMA

*TAPSE, tricuspid annular plane systolic excursion; GA, Gestational age (weeks); PMA, postmenstrual age (weeks); E', early diastolic velocity; A', late diastolic velocity; S', systolic velocity*.

### BPD vs. No-BPD Group

As previously mentioned, TAPSE and TDI-derived velocities were influenced by GA and PMA; therefore, we included both GA and PMA in the subsequent analysis.

We found a negative correlation between the plasma NTproBNP levels and TAPSE (Spearman's Rho = −0.36, *p* = 0.0001), with similar results regarding diastolic velocities derived of TDI (data not shown).

In the multivariate study, echocardiography together with NTproBNP levels at 14 days of life demonstrated good diagnostic accuracy indices (Table 4). The model that included NTproBNP at 14 DOL, TAPSE, GA and PMA had an area under curve (AUC) of 0.988 (95% CI 0.97–1), a sensitivity (Se) of 68.57% and a specificity (Sp) of 97.51%. See Table 5 for more details regarding the two selected models. Both models are reliable with a loss of prediction (shrinkage) of 0.15% estimated for the model that includes TAPSE, NTproBNP at14 DOL, GA, and PMA, and a shrinkage of 6.54% for the model including also E' and A'. Moreover, both models are consistent as shown by a mean pseudo *R*^2^ of 0.68 and 0.75, respectively.

**Table 4:** Goodness of fit parameters of different BPD predictive models.

Included variables^*^	**AUC**	**Se**	**Sp**	**AIC**	**BIC**
proBNP14	0.976	60.0	99.03	72.3	79.1
proBNP14 + TAPSE	0.988	68.57	97.51	51.1	68.1
proBNP14 + E'	0.984	75.0	97.04	51	68
proBNP14 + A'	0.99	80.56	97.04	49.3	66.3
proBNP14 + TAPSE + E'	0.99	81.82	97.98	50.3	70.7
proBNP14 + TAPSE + A'	0.99	84.85	98.48	50.2	70.6
proBNP14 +TAPSE + E'+ A'	0.99	90.91	98.99	44.1	67.9

proBNP14, logarithmic of NTproBNP at 14 days of life; TAPSE, tricuspid annular plane systolic excursion; E', early diastolic velocity; A', late diastolic velocity; AUC, area under the curve; Se, sensibility; Sp, specificity; AIC, Akaike information criterion; BIC, Bayesian information criterion.

* *Gestational age and postmenstrual age had been included in the predictive models*.

**Table 5:** Parameters of the selected BPD predictive models.

	**Coefficient (CI 95%)**	***p***	**Coefficient (CI 95%)**	***p***
TAPSE	−5.74 (−10.95 to −0.52)	0.031	−6.68 (−13.14 to −0.23)	0.042
proBNP14	7.26 (3.74–10.79)	0.0001	10.82 (5.05–16.58)	0.0001
GA	−1.26 (−1.95 to −0.58)	0.0001	−1.60 (−2.49 to −0.71)	0.0001
PMA	0.38 (0.01–0.75)	0.045	0.31 (−0.12 to −0.75)	0.16
E'			0.76 (−0.08 to −1.61)	0.075
A'			−0.64 (−1.20 to −0.087)	0.023
Multilevel mixed-effects logistic regression: Log Likelihood = −12.12 Wald Chi^2^ (4) = 223.41; *p* = 0.001; Covar (cc) = 1.71e−24			Multilevel mixed-effects logistic regression: Log Likelihood = −8.67 Wald Chi^2^ (6) = 217.15; *p* = 0.001; Covar (cc) = 3.89e−25	
External validation: Shrinkage = 0.15% Internal validation: Cross-validation: mean pseudo *R*^2^ = 0.68			External validation: Shrinkage = 6.54% Internal validation: Cross-validation: mean pseudo *R*^2^ = 0.75	

*TAPSE, tricuspid annular plane systolic excursion; proBNP14, logarithmic of NTproBNP at 14 days of life; GA, gestational age; PMA, Postmenstrual age; E', early diastolic velocity; A', late diastolic velocity; Covar, covariance*.

## Discussion

This study has allowed us to describe normative data for the TAPSE and TDI velocities in VLBWIs and to show how myocardial maturation (measured by TnEcho and NTproBNP at 14 DOL) is different in VLBWIs that develop BPD compared to in those who do not develop BPD. We have shown how both birth GA and postnatal life impact TAPSE and TDI velocities and are important factors to consider when performing TnEcho in VLBWIs.

The myocardium of the VLBWI has different diastolic properties than that of the term newborn, with fewer contractile fibers, which is reflected in deficient passive ventricular filling and a more important contribution of atrial contraction (28). This results in a higher A' wave than E' wave, similar to what is found during fetal life (29). In addition, TAPSE and RV TDI velocities were lower in VLBWIs with lower GA, according to what was previously reported (22, 30). Our measures were similar to those described by Murase et al. (31) although a complete comparison of our data with theirs is not possible since they don't report numeric data. Also partially similar but inferior to those described by Ciccone et al. (32), they included preterm infants of greater gestational age. Moreover, we have also found, in accordance with the previous literature, a decrease in RV MPI-TDI over time (19).

Interestingly, as mentioned before, both GA and chronological age influence TnEcho parameters in VLBWIs. In children, TDI increases mostly related to age but also to body surface and heart rate (33). Saleemi et al. (10) reported an increase in left ventricle TDI measures during the first month of life in preterm infants <32 weeks of GA. Murase et al. (19) evaluated the evolution of TDI measures in the first week of life in VLBWIs. Our study was conducted over a longer study period and on a higher number of TnECHO cases, with our patients followed weekly until 36 weeks of PMA. Di Maria et al. (6) found similar results when comparing TnEcho at 7 DOL and at 36 weeks of PMA, they observed how GA influences the results at 7 DOL but not at 36 weeks of PMA. TDI measures in VLBWIs at the term-corrected age seem to be higher than those in term newborns at 48 h of life (21). Nevertheless, they found similar left ventricle function to that of term newborns after the transitional period (34). Eriksen et al. (35) described differences in late preterm TDI measures from the transitional period to the term-corrected age. Interestingly, normative TDI values in term newborns and in VLBWIs have been based on studies performed during the transitional period (26). Not only the chronology of the assessments make a difference in the compared studies but it is also important to remark that we excluded those preterm infants who were IUGR or had HSPDA or BPD when estimating the normative values while the referred studies use different inclusion criteria (19, 21, 31). This heterogenicity makes the values of TnEcho parameters not easy to compare.

There are only a few studies that explore TAPSE in VLBWIs. There is normative data on normal values of TAPSE in healthy babies and newborns adjusted for age and body surface area (7, 36), but data in VLBWIs are lacking. We found similar results to those reported by Koestenberger et al. (22), who published TAPSE reference values in VLBWIs by GA in the first 48 h of life. We have demonstrated how TAPSE values are influenced by GA and chronological age, and we report our results taking both into account, not only for TAPSE but also for the MPI-TDI and RV TDI velocities. We have provided easy-to-use percentile tables and figures that we think could be helpful when performing TnEcho in the NICU.

Myocardial function maturation in VLWBIs that develop BPD seems to be delayed. A previous study from our group showed how NTproBNP levels at 14 days of life were increased in those who developed BPD with an optimal cut-off value of 2,264 pg/ml (AUC 0.93; Se 100%; and Sp 86%) (15). Adding biochemical and echocardiographic markers as soon as 14 DOL could help identify VLBWIs at a higher risk of developing BPD. A delay in the MPI decrease measured by a power Doppler has been reported (4, 37). TAPSE values have been related to early pulmonary hypertension in VLBWIs (38), but the association with the later development of BPD has not been previously explored. When combining NTproBNP levels and TnEcho in the assessment of VLBWI cardiac function, we found that RV echocardiographic parameters, GA and PMA, when added to NTproBNP levels measured at 14 DOL, reach high diagnostic accuracy indices. The correlation of NTproBNP plasma levels with TDI has been previously explored in adults, children and, recently, in VLBWIs with and without PDA (39), showing a negative correlation between the NTproBNP plasma levels. Our study has similar results and show how TAPSE and NTproBNP plasma levels are also associated. In adults, the natriuretic peptide combined with TDI is used in heart failure evaluations and when considering dyspnea in a differential diagnosis (40). In children, the combined biochemical plus echocardiographic cardiac evaluation could be useful to predict anthracycline-induced cardiotoxicity (41). In infants aged 6–12 months with BPD, BNP, and echocardiography have been explored, with no significant differences (42). Behere et al. have observed that the increase in BNP and the dilatation of right cavities can predict the risk of pulmonary hypertension in infants with BPD; the authors did not observe consistent results with TAPSE or TDI (43). To our knowledge, this is the first study to evaluate NTproBNP levels, TAPSE values and RV TDI velocities in VLBWIs related to the development of BPD. Using both biochemical and echocardiographic biomarkers in the clinical settings could be suggested for the identification of VLBWI with a higher risk of developing BPD. This approach might help develop new therapeutic/prophylactic strategies in this high risk population. NTproBNP levels are easy to obtain from a routine blood sample and are available in a short period of time and, on the other hand, TnEcho could be performed in most NICUs, it is safe (44) and reproducible (27). This makes both techniques widely available and ready to use in routine clinical practice.

We have explored different predictive models with very close diagnostic accuracy indices. We suggest including TAPSE since it is an easy measurement in TnEcho and has good interobserver and intraobserver reproducibility and reliability (27). Acquiring TAPSE is easy and fast and can be measured in non-collaborating patients and even with suboptimal images, making it perfect for use in VLBWIs. In addition, TAPSE is measured by M-mode, a conventional ultrasound mode that can be available with no need for expensive equipment. A modified TAPSE measured, subcostal TAPSE (S-TAPSE), has been suggested as an useful alternative in patients with an inadequately visualized four chambers view and a good correlation with TAPSE has been described in children and neonates (45).

Our study has several limitations that need to be addressed. While this study has a good sample size compared to those of other studies, a larger sample size, including patients born before 25 weeks of GA, would strengthen our results. Nevertheless, the obtained models have proven to have good internal and external validity, thus could be considered reliable and reproducible. In the BPD group, the existence of pulmonary hypertension has not been evaluated, and this factor could influence the results. To define normative values of echocardiographic parameters, a subpopulation of VLBWIs were selected, excluding those with BPD, with hemodynamically significant PDA or that were small of gestation age. Nevertheless, other exclusion criteria could be suggested, such as ventilatory support. A detailed description of the ventilatory parameters that each patient had while performing the TnEcho is lacking in our study but TnEcho was always performed on good respiratory condition with no major events happening as previously reported (27). However, ventilatory support has not been found to be correlated with TAPSE (46). As stated before we would only perform the echocardiographic exams if the VLBWIs were stable, therefore none of them had hypotension or need for inotropic supports during a TnEcho. Thus, our results might not be extrapolated to VLBWIs with hemodynamic instability.

Our findings suggest that the myocardial function maturation of VLBWIs depends both on preterm birth itself and on early extrauterine life exposure. In considering GA and PMA when performing TnEcho in the VLBWI, we suggest that TnEcho paired with NTproBNP could be useful for cardiac assessment in this population and could help identify which of them are at a higher risk of BPD and would potentially benefit from preventive strategies.

## Data Availability Statement

The raw data supporting the conclusions of this article will be made available by the authors, without undue reservation, to any qualified researcher.

## Ethics Statement

The studies involving human participants were reviewed and approved by Comité de Ética de la Investigación de Cádiz. Written informed consent to participate in this study was provided by the participants' legal guardian/next of kin.

## Author Contributions

PM-A, PZ-R, IB-F, and SL-L contributed conception and design of the study. PM-A and PZ-R organized the database. IB-F performed the statistical analysis. PM-A wrote the first draft of the manuscript. PM-A and IB-F wrote sections of the manuscript. All authors contributed to manuscript revision, read, and approved the submitted version.

### Conflict of Interest

The authors declare that the research was conducted in the absence of any commercial or financial relationships that could be construed as a potential conflict of interest.

## Abbreviations

BPD: Bronchopulmonary dysplasia
VLBWIs: very-low-birth-weight infants
TnEcho: Targeted neonatal echocardiography
TDI: tissue Doppler imaging
PDA: patent ductus arteriosus
NTproBNP: N-terminal pro B type natriuretic peptide
GA: gestational age
PMA: postmenstrual age
RV: right ventricle
TAPSE: tricuspid annular plane systolic excursion
E': Early diastolic velocity
A': late diastolic velocity
S': systolic velocity
MPI-TDI: The myocardial performance index measured by TDI
DOL: days of life.
