# Supplementary material for: Myocardial Function Maturation in Very-Low-Birth-Weight Infants and Development of Bronchopulmonary Dysplasia
Source: Front Pediatr. 2020 Jan 17;7:556. doi: 10.3389/fped.2019.00556 (PMC6978685; doi:10.3389/fped.2019.00556)
Supplement: Supplementary file 1 [file Table_1.DOCX]

**Table S1.** TAPSE and right ventricle TDI derived parameters in the first two month of life by gestational age.

| DOL | 25-27 GA | | | | 28-30 GA | | | | 31-32 GA | | | |
| --- | --- | --- | --- | --- | --- | --- | --- | --- | --- | --- | --- | --- |
|  | E´ | A´ | TAPSE | S´ | E´ | A´ | TAPSE | S´ | E´ | A´ | TAPSE | S´ |
| 1 | 4.03± 0.92 | 8.32± 0.77 | 0.52±0.06 | 4.57± 0.52 | 4.69± 1.05 | 8.50±1.57 | 0.67± 0.09 | 5.48± 0.86 | 4.98± 0.82 | 8.66± 1.13 | 0.66±0.12 | 5.53± 0.55 |
| 3 | 5.01± 0.61 | 6.42± 0.26 | 0.68±0.23 | 6.32± 1.71 | 5.10± 1.87 | 8.56±1.42 | 0.70± 0.11 | 6.08± 1.08 | 4.86± 0.35 | 9.28± 1.58 | 0.67±0.06 | 6.02± 0.56 |
| 7 | 6.14± 1.63 | 9.68± 2.22 | 0.61±0.08 | 6.24± 0.75 | 5.85± 1.90 | 10.09±1.73 | 0.72± 0.10 | 6.20± 0.75 | 5.47± 1.22 | 9.49± 1.25 | 0,78±0.14 | 5.96± 0.73 |
| 14 | 5.24± 0.97 | 9.41± 3.30 | 0.77±0.06 | 5.49± 1.32 | 6.32± 1.49 | 10.73±1.61 | 0.75± 0.13 | 6.74± 1.00 | 6.53± 1.12 | 9.98± 1.73 | 0,83±0.15 | 6.31± 1.11 |
| 21 | 4.91± 0.94 | 10.02± 2.50 | 0.78±0.08 | 6.12± 1.15 | 6.92± 1.62 | 10.86± 2.20 | 0.85± 0.10 | 6.87± 1.44 | 8.15± 1.18 | 11.86± 2.75 | 1.01±0.21 | 8.10± 1.20 |
| 28 | 5.88± 0.71 | 10.06± 0.52 | 0.85±0.12 | 7.00± 0.83 | 7.12± 1.83 | 11.03± 1.89 | 0.94± 0.11 | 6.99± 0.97 | 6.81± 0.90 | 9.00± 3.45 | 0.99±0.11 | 6.36± 1.23 |
| 35 | 7.37± 2.00 | 10.84± 1.09 | 0.93±0.10 | 6.85± 1.62 | 6.80± 1.59 | 11.69± 2.08 | 1.01± 0.14 | 7.43± 1.06 | 9.63 | 11.90 | 1.11 | 8.82 |
| 42 | 7.25± 0.92 | 10.76± 1.98 | 0.90±0.14 | 7.71± 0.80 | 7.32± 1.33 | 12.57± 2.06 | 1.11± 0.14 | 7.71± 1.39 |  |  |  |  |
| 49 | 6.29± 2.09 | 12.10± 1.07 | 0.93±0.20 | 7.30± 0.60 | 8.72± 1.49 | 11.68± 2.01 | 1.09± 0.12 | 8.21± 0.91 |  |  |  |  |
| 56 | 7.05± 0.70 | 11.80± 1.11 | 0.98±0.01 | 7.19± 0.50 | 6.98± 2.61 | 9.91± 1.12 | 1.21± 0.13 | 7.62± 2.12 |  |  |  |  |

DOL: days of life. TAPSE: tricuspid annular plane systolic excursion. E′: early diastolic velocity. A′: late diastolic velocity. S´: systolic velocity. GA: Gestational age (weeks), horizontal line.
