# Supplementary material for: Myocardial Function Maturation in Very-Low-Birth-Weight Infants and Development of Bronchopulmonary Dysplasia
Source: Front Pediatr. 2020 Jan 17;7:556. doi: 10.3389/fped.2019.00556 (PMC6978685; doi:10.3389/fped.2019.00556)
Supplement: Supplementary file 2 [file Table_2.DOCX]

**Table S2.** Observed and predicted TAPSE and right ventricle TDI velocities by gestational age and postmenstrual age.

| **observed TAPSE** | | | |  |  |  |  |  |  | **predicted TAPSE** | | | |  |  |  |  |  |
| --- | --- | --- | --- | --- | --- | --- | --- | --- | --- | --- | --- | --- | --- | --- | --- | --- | --- | --- |
| PMA | GA | 26 | 27 | 28 | 29 | 30 | 31 | 32 |  | PMA | GA | 26 | 27 | 28 | 29 | 30 | 31 | 32 |
| 26 | | 0.59 |  |  |  |  |  |  |  | 26 | | 0.55 |  |  |  |  |  |  |
| 27 | | 0.54 | 0.51 |  |  |  |  |  |  | 27 | | 0.62 | 0.59 |  |  |  |  |  |
| 28 | | 0.7 | 0.64 | 0.61 |  |  |  |  |  | 28 | | 0.68 | 0.65 | 0.62 |  |  |  |  |
| 29 | | 0.85 | 0.82 | 0.74 | 0.67 |  |  |  |  | 29 | | 0.75 | 0.72 | 0.69 | 0.66 |  |  |  |
| 30 | | 0.63 | 0.71 | 0.72 | 0.71 | 0.73 |  |  |  | 30 | | 0.81 | 0.78 | 0.75 | 0.72 | 0.69 |  |  |
| 31 | | 0.9 | 0.9 | 0.77 | 0.70 | 0.74 | 0.64 |  |  | 31 | | 0.88 | 0.85 | 0.82 | 0.79 | 0.76 | 0.73 |  |
| 32 | | 0.87 | 0.86 | 0.90 | 0.89 | 0.82 | 0.80 | 0.77 |  | 32 | | 0.94 | 0.91 | 0.88 | 0.85 | 0.82 | 0.79 | 0.76 |
| 33 | | 0.87 | 0.90 | 0.98 | 0.93 | 0.86 | 0.85 | 0.66 |  | 33 | | 1.01 | 0.98 | 0.95 | 0.92 | 0.89 | 0.86 | 0.83 |
| 34 | | 0.98 | 0.87 | 1.05 | 1.00 | 0.99 | 1.01 | 0.80 |  | 34 | | 1.07 | 1.04 | 1.01 | 0.98 | 0.95 | 0.92 | 0.89 |
| 35 | | 0.87 | 0.99 | 1.12 | 1.09 | 1.05 | 0.99 |  |  | 35 | | 1.14 | 1.11 | 1.08 | 1.05 | 1.02 | 0.99 | 0.96 |
| 36 | |  | 0.67 | 1.21 | 1.07 | 1.18 | 1.11 |  |  | 36 | | 1.20 | 1.17 | 1.50 | 1.11 | 1.08 | 1.05 | 1.02 |
|  | |  |  |  |  |  |  |  |  |  | |  |  |  |  |  |  |  |
| **observed E´** | | |  |  |  |  |  |  |  | **predicted E´** | | |  |  |  |  |  |  |
| PMA | GA | 26 | 27 | 28 | 29 | 30 | 31 | 32 |  | PMA | GA | 26 | 27 | 28 | 29 | 30 | 31 | 32 |
| 26 | | 5.01 |  |  |  |  |  |  |  | 26 | | 4.59 |  |  |  |  |  |  |
| 27 | | 5.21 | 3.36 |  |  |  |  |  |  | 27 | | 4.97 | 4.81 |  |  |  |  |  |
| 28 | | 5.92 | 5.12 | 5.14 |  |  |  |  |  | 28 | | 5.34 | 5.18 | 5.02 |  |  |  |  |
| 29 | | 5.77 | 4.41 | 5.76 | 4.64 |  |  |  |  | 29 | | 5.72 | 5.56 | 5.4 | 5.24 |  |  |  |
| 30 | | 5.71 | 4.81 | 7.04 | 6.05 | 4.49 |  |  |  | 30 | | 6.09 | 5.93 | 5.77 | 5.61 | 5.46 |  |  |
| 31 | | 6.27 | 5.71 | 6.57 | 5.91 | 5.61 | 4.81 |  |  | 31 | | 6.47 | 6.31 | 6.15 | 5.99 | 5.84 | 5.68 |  |
| 32 | | 6.52 | 6.67 | 7.93 | 7.14 | 6.50 | 5.47 | 6.12 |  | 32 | | 6.84 | 6.68 | 6.52 | 6.36 | 6.21 | 6.05 | 5.89 |
| 33 | | 6.62 | 6.97 | 5.63 | 6.61 | 6.93 | 6.63 | 5.41 |  | 33 | | 7.22 | 7.06 | 6.90 | 6.74 | 6.59 | 6.43 | 6.27 |
| 34 | | 7.72 | 4.71 | 7.55 | 7.17 | 7.20 | 8.15 | 5.61 |  | 34 | | 7.59 | 7.43 | 7.27 | 7.11 | 6.96 | 6.80 | 6.64 |
| 35 | | 6.42 | 7.12 | 9.26 | 7.43 | 7.36 | 6.81 |  |  | 35 | | 7.97 | 7.81 | 7.65 | 7.49 | 7.34 | 7.18 | 7.02 |
| 36 | |  | 5.41 | 6.98 | 8.45 | 6.92 | 9.63 |  |  | 36 | | 8.34 | 8.18 | 8.02 | 7.86 | 7.71 | 7.55 | 7.39 |
|  | |  |  |  |  |  |  |  |  |  | |  |  |  |  |  |  |  |
| **observed A´** | | |  |  |  |  |  |  |  | **predicted A´** | | |  |  |  |  |  |  |
| PMA | GA | 26 | 27 | 28 | 29 | 30 | 31 | 32 |  | PMA | GA | 26 | 27 | 28 | 29 | 30 | 31 | 32 |
| 26 | | 7.91 |  |  |  |  |  |  |  | 26 | | 8.47 |  |  |  |  |  |  |
| 27 | | 8.58 | 8.52 |  |  |  |  |  |  | 27 | | 8.91 | 8.71 |  |  |  |  |  |
| 28 | | 6.66 | 11.06 | 8.77 |  |  |  |  |  | 28 | | 9.34 | 9.14 | 8.88 |  |  |  |  |
| 29 | | 9.21 | 11.8 | 10.81 | 8.46 |  |  |  |  | 29 | | 9.77 | 9.57 | 9.31 | 10.08 |  |  |  |
| 30 | | 9.78 | 8.41 | 12.23 | 10.00 | 8.42 |  |  |  | 30 | | 10.21 | 10.02 | 9.75 | 10.52 | 9.27 |  |  |
| 31 | | 9.88 | 10.17 | 9.98 | 9.66 | 9.67 | 8.65 |  |  | 31 | | 10.64 | 10.44 | 10.18 | 10.95 | 9.72 | 9.49 |  |
| 32 | | 9.13 | 11.25 | 11.74 | 10.89 | 11.36 | 9.22 | 8.72 |  | 32 | | 11.07 | 10.87 | 10.61 | 11.38 | 10.15 | 9.92 | 9.69 |
| 33 | | 11.45 | 12.90 | 10.06 | 11.07 | 11.57 | 10.10 | 9.42 |  | 33 | | 11.51 | 11.32 | 11.05 | 11.82 | 10.59 | 10.36 | 10.13 |
| 34 | | 10.80 | 12.00 | 11.27 | 12.14 | 10.46 | 11.86 | 7.32 |  | 34 | | 11.94 | 11.74 | 11.48 | 12.25 | 11.02 | 10.79 | 10.56 |
| 35 | | 8.72 | 13.00 | 10.25 | 12.71 | 12.60 | 9.00 |  |  | 35 | | 12.38 | 12.18 | 11.92 | 12.69 | 11.46 | 11.23 | 11.00 |
| 36 | | 9.42 |  | 9.91 | 12.40 | 13.63 | 11.90 |  |  | 36 | | 12.81 | 12.61 | 12.35 | 13.12 | 11.89 | 11.66 | 11.43 |
|  | |  |  |  |  |  |  |  |  |  | |  |  |  |  |  |  |  |
| **observed S´** | | |  |  |  |  |  |  |  | **predictive S´** | | |  |  |  |  |  |  |
| PMA | GA | 26 | 27 | 28 | 29 | 30 | 31 | 32 |  | PMA | GA | 26 | 27 | 28 | 29 | 30 | 31 | 32 |
| 26 | | 5.21 |  |  |  |  |  |  |  | 26 | | 5.44 |  |  |  |  |  |  |
| 27 | | 5.21 | 4.31 |  |  |  |  |  |  | 27 | | 5.66 | 5.55 |  |  |  |  |  |
| 28 | | 7.42 | 6.72 | 5.82 |  |  |  |  |  | 28 | | 6.06 | 5.86 | 5.67 |  |  |  |  |
| 29 | | 6.47 | 4.67 | 6.38 | 5.46 |  |  |  |  | 29 | | 6.36 | 6.16 | 5.97 | 5.78 |  |  |  |
| 30 | | 6.22 | 6.42 | 7.72 | 6.18 | 5.36 |  |  |  | 30 | | 6.67 | 6.47 | 6.28 | 6.09 | 5.90 |  |  |
| 31 | | 7.82 | 6.72 | 5.67 | 6.30 | 6.10 | 5.40 |  |  | 31 | | 6.98 | 6.78 | 6.59 | 6.40 | 6.21 | 6.01 |  |
| 32 | | 8.82 | 7.02 | 7.01 | 7.32 | 6.92 | 6.02 | 6.42 |  | 32 | | 7.28 | 7.08 | 6.89 | 6.70 | 6.51 | 6.31 | 6.12 |
| 33 | | 7.72 | 7.17 | 6.38 | 7.17 | 7.16 | 5.84 | 5.92 |  | 33 | | 7.59 | 7.39 | 7.02 | 7.01 | 6.82 | 6.62 | 6.43 |
| 34 | | 7.12 | 7.17 | 7.25 | 7.93 | 6.76 | 6.48 | 4.51 |  | 34 | | 7.90 | 7.70 | 7.51 | 7.32 | 7.13 | 6.93 | 6.74 |
| 35 | | 7.42 | 7.72 | 7.57 | 8.70 | 7.68 | 8.10 |  |  | 35 | | 8.21 | 8.01 | 7.82 | 7.63 | 7.44 | 7.24 | 7.05 |
| 36 | |  | 6.92 | 7.62 | 8.52 | 6.52 | 6.36 |  |  | 36 | | 8.51 | 8.31 | 8.12 | 7.93 | 7.74 | 7.54 | 7.35 |

TAPSE: tricuspid annular plane systolic excursion. E′: early diastolic velocity. A′: late diastolic velocity. S´: systolic velocity. GA: Gestational age (weeks), horizontal line. PMA: postmenstrual age (weeks), vertical line.
